# Attenuation of choroidal neovascularization by dietary intake of ω-3 long-chain polyunsaturated fatty acids and lutein in mice

**DOI:** 10.1371/journal.pone.0196037

**Published:** 2018-04-25

**Authors:** Ryoji Yanai, Shang Chen, Sho-Hei Uchi, Tomoaki Nanri, Kip M. Connor, Kazuhiro Kimura

**Affiliations:** 1 Department of Ophthalmology, Yamaguchi University Graduate School of Medicine, Ube, Yamaguchi, Japan; 2 Santen Pharmaceutical Co. Ltd., Osaka, Japan; 3 Angiogenesis Laboratory, Department of Ophthalmology, Massachusetts Eye and Ear Infirmary, Harvard Medical School, Cambridge, Massachusetts, United States of America; University of Florida, UNITED STATES

## Abstract

Dietary ω-3 long-chain polyunsaturated fatty acids (LCPUFAs) and lutein each protect against age-related macular degeneration (AMD). We here examined the effects of ω-3 LCPUFAs and lutein supplementation in a mouse model of AMD. Mice were assigned to four groups: (1) a control group fed an ω-3 LCPUFA–free diet, (2) a lutein group fed an ω-3 LCPUFA–free diet with oral administration of lutein, (3) an ω-3 group fed an ω-3 LCPUFA–supplemented diet, and (4) an ω-3 + lutein group fed an ω-3 LCPUFA–supplemented diet with oral administration of lutein. Mice were fed the defined diets beginning 2 weeks before, and received lutein with an oral gavage needle beginning 1 week before, induction of choroidal neovascularization (CNV) by laser photocoagulation. The area of CNV measured in choroidal flat-mount preparations was significantly reduced in mice fed ω-3 LCPUFAs or lutein compared with those in the control group, and it was reduced in an additive manner in those receiving both ω-3 LCPUFAs and lutein. The concentrations of various inflammatory mediators in the retina or choroid were reduced in mice fed ω-3 LCPUFAs or lutein, but no additive effect was apparent. The generation of reactive oxygen species (ROS) in chorioretinal lesions revealed by dihydroethidium staining as well as the expression of NADPH oxidase 4 (Nox4) in the retina revealed by immunohistofluorescence and immunoblot analyses were attenuated by ω-3 LCPUFAs and lutein in a synergistic manner. Our results thus show that dietary intake of ω-3 LCPUFAs and lutein attenuated CNV in an additive manner and in association with suppression of inflammatory mediator production, ROS generation, and Nox4 expression. Dietary supplementation with both ω-3 LCPUFAs and lutein warrants further study as a means to protect against AMD.

## Introduction

Age-related macular degeneration (AMD) is the leading cause of blindness in developed countries. There are two subtypes of this condition: neovascular (wet) AMD and dry AMD [1–3]. The inadequacy of current treatments for AMD has focused attention on preventive approaches [4, 5]. Clinical studies of such approaches have included the Age-Related Eye Disease Study (AREDS) [6] and AREDS2 [7], the former of which identified beneficial effects of a multivitamin supplement and zinc, and the latter of which found that lutein, a xanthophyll carotenoid and antioxidant nutrient supplement [8], provided additional protection.

Lutein is a major component of macular pigment in the human retina and protects against light-induced retinal damage through absorption of blue light and neutralization of reactive oxygen species (ROS) [9]. Although AMD is a multifactorial and complex disease, oxidative stress is a major risk factor in its pathogenesis. Lutein supplementation was shown to increase the serum lutein concentration as well as to improve the visual sensitivity of individuals with early-stage AMD [10], and it is thought to hold promise as a prophylactic regimen [8, 11–14].

We previously showed that ω-3 long-chain polyunsaturated fatty acids (ω-3 LCPUFAs), essential dietary fatty acids [15] that are highly enriched in the retina [16], are potent inhibitors of intraocular neovascularization in mice [17]. We have now investigated the effects of dietary intake of both ω-3 LCPUFAs and lutein on the development of laser-induced choroidal neovascularization (CNV) in a mouse model of neovascular AMD.

## Methods

### Animals, diets, and anesthesia

**Fig 1:**
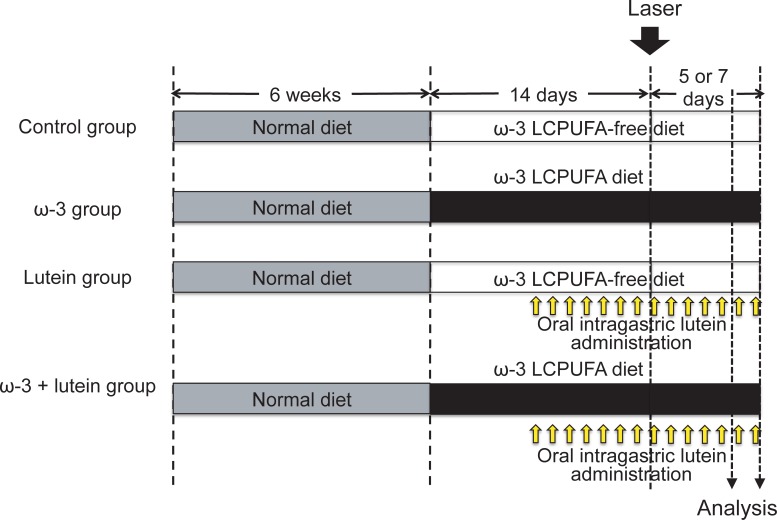
Experimental design. Mice maintained on a normal diet up to 6 weeks of age were then fed a diet either supplemented with ω-3 LCPUFAs (*n* = 80) or free of ω-3 LCPUFAs (*n* = 80) beginning 2 weeks before the induction of CNV. Half of the mice on each diet received lutein daily via an oral gavage needle beginning 1 week before the induction of CNV. The animals were killed 7 days after laser photocoagulation for measurement of CNV size (*n* = 15 for each group [*n* = 5 for each of three separate experiments), serum fatty acid and lutein concentrations (*n* = 5 for each group from among the 15 mice examined for measurement of CNV size, and one sample was used for the calibration of gas chromatography), and cytokine and chemokine concentrations in the retina and choroid (*n* = 5 for each group) as well as for immunoblot analysis of Nox4 (*n* = 5 for each group) and immunohistofluorescence analysis of Nox4 (*n* = 5 for each group). For detection of ROS (*n* = 10 for each group [*n* = 5 for each of two separate experiments]), the animals were killed 5 days after laser photocoagulation.

Six-week-old male C57BL/6 mice (*n* = 160) that had been maintained on a normal diet were obtained from Chiyoda Kaihatsu (Tokyo, Japan). The mice were assigned to four groups: (1) a control group fed an ω-3 LCPUFA–free diet, (2) a lutein group fed an ω-3 LCPUFA–free diet with oral administration of lutein, (3) an ω-3 group fed an ω-3 LCPUFA–supplemented diet, and (4) an ω-3 + lutein group fed an ω-3 LCPUFA–supplemented diet with oral administration of lutein. The animals were thus transferred to a diet that was either supplemented with or free of ω-3 LCPUFAs (Fig 1), as described previously [18] (Oriental Yeast, Tokyo, Japan). In addition, mice received 0.055 ml (0.5 mg/kg) of lutein (Koyojapan, Tokyo, Japan) or vehicle (control and ω-3 groups) via an oral gavage needle daily beginning 1 week after the onset of feeding with either defined diet.

Mice were anesthetized for the laser photocoagulation procedure by intraperitoneal injection of a mixture of ketamine (90 mg/kg) (Daiichi Sankyo, Tokyo, Japan) and xylazine (10 mg/kg) (Bayer Yakuhin, Osaka, Japan), and their pupils were dilated by topical instillation of 0.5% tropicamide and 0.5% phenylephrine hydrochloride ophthalmic solutions (Santen, Osaka, Japan). Animals were treated in accordance with the ARVO Statement for the Use of Animals in Ophthalmic and Vision Research, and the study was approved by the Animal Care and Use Committee of Yamaguchi University (approval no. 41–032).

### Laser-induced CNV

Forty mice of each group were subjected to laser photocoagulation in both eyes at 2 weeks after the onset of feeding with the defined diets. The procedure was performed as described previously [18] with settings including a spot size of 75 μm, pulse duration of 0.1 s, and laser power of 200 mW. One investigator (S.C.) executed all laser photocoagulation.

### Choroidal flat-mount preparation

For evaluation of the size of CNV lesions (four laser spots per eye), 15 mice (*n* = 5 for each of three separate experiments) of each group were killed by cervical dislocation at 7 days after laser photocoagulation. The eyes were enucleated and fixed in 4% paraformaldehyde for 60 min at room temperature, the cornea and lens were removed, and the entire retina was carefully dissected from the eyecup. Radial cuts (average of eight) were made from the edge of the eyecup to the equator, and the preparation was then washed with ice-cold phosphate-buffered saline containing 0.3% Tween 20. For visualization of vessels, the tissue was stained overnight at room temperature with Alexa Fluor 488–conjugated *Griffonia* (*Bandeiraea*) *simplicifolia* isolectin B4 (Invitrogen, Carlsbad, CA) at a dilution of 1:100. The preparation was then flat-mounted and imaged as described previously [17]. The CNV area was measured with the use of Image J software 1.50 (National Institutes of Health, Bethesda, MD).

### Analysis of fatty acids and lutein in serum

Retro-orbital sinus blood was collected rapidly after removal of the eyeballs from mice for choroidal flat-mount preparation as described above. The blood samples were collected for preparation of serum as described previously [17]. Serum was analyzed for lutein content and the fatty acid composition of lipids by gas chromatography at SRL Clinical Laboratory (Tokyo, Japan).

### Analysis of cytokines and chemokines in the retina and choroid

Eyeballs removed 7 days after CNV induction (10 laser spots per eye) were trimmed of extraocular muscles and soft tissue. The retina and choroid were isolated and were separately pooled (*n* = 6 to 10) to reduce biological variability. The tissue was homogenized in 0.1 ml per eye of a lysis buffer containing protease inhibitors (Roche Diagnostics, Indianapolis, IN), and the homogenate was centrifuged at 16,200 × *g* for 10 min at 4°C. The protein concentration of the resulting supernatant was determined with a DC (detergent-compatible) protein assay (Bio-Rad, Hercules, CA), and the supernatant was then stored at –80°C until assay of cytokine and chemokine concentrations with the use of a Bio-Plex Pro Mouse Cytokine 23-Plex Panel and Bio-Plex Manager software version 4.1.1 (Bio-Rad).

### ROS detection in retina-choroid sections

Generation of ROS in eyeballs removed 5 days after CNV induction (six laser spots per eye) was detected by staining with dihydroethidium (DHE) as described previously [19]. Eyes were embedded in optimum cutting temperature (OCT) compound (Thermo Fisher Scientific, Waltham, MA) and subjected to rapid freezing immediately after enucleation. Cryosections of the retinal pigment epithelium (RPE)–choroid were prepared at a thickness of 14 μm. Confocal images of DHE fluorescence were acquired with a BZ-X700 All-in-One Microscope (Keyence, Osaka, Japan) equipped with a 20× objective and 530-nm excitation and 615-nm emission filters. All images were collected at the same photomultiplier tube voltage, gain, and offset and were converted to gray scale with the use of Photoshop Element 15 (Adobe, San Jose, CA). The RPE and choroid were examined, with the sclera and extraocular tissues being excluded from analysis. The sum of the gray values of all pixels (integrated density) per CNV area of interest was measured with Image J software 1.50.

### Immunofluorescence microscopy

Eyes removed 7 days after CNV induction (six laser spots per eye) were processed as described above for preparation of cryosections (thickness, 12 μm) of the RPE-choroid. The sections were fixed in 4% paraformaldehyde, washed with Ca^2+^- and Mg^2+^-free phosphate-buffered saline [PBS(–)], and incubated for 15 min at room temperature in PBS(–) containing 2% fetal bovine serum (FBS) and 0.3% Triton X-100. They were then incubated for 16 h at 4°C with antibodies to NADPH oxidase 4 (Nox4) (Abcam, Cambridge, UK) at a 1:100 dilution in PBS(–) containing 2% FBS and 0.3% Triton X-100, washed with PBS(–), incubated for 1 h at 4°C with Alexa Fluor 647–conjugated secondary antibodies (Invitrogen) at a 1:1000 dilution in PBS(–) containing 2% FBS and 0.3% Triton X-100, and washed again with PBS(–). The sections were finally mounted with Vectashield (Vector Laboratories, Burlingame, CA), and confocal images were acquired as described above for DHE staining.

### Immunoblot analysis

Retinas isolated from eyes 7 days after CNV induction (10 laser spots per eye) were pooled (*n* = 6 to 10) to reduce biological variability. The tissue was homogenized and the homogenate was centrifuged as described above, and portions of the supernatant (20 μg of protein) were fractionated by SDS-polyacrylamide gel electrophoresis on a 4% to 20% gradient gel (Invitrogen). The separated proteins were transferred to a polyvinylidene difluoride membrane (Millipore, Billerica, MA), which was then incubated with StartingBlock Blocking Buffer (Thermo Fisher Scientific) before exposure overnight at 4°C to rabbit polyclonal antibodies to Nox4 (1:1000 dilution) (Abcam) or to β-actin (1:2000 dilution) (Cell Signaling Technology, Danvers, MA). The membrane was then washed three times (5 min each time) with Tris-buffered saline containing 0.5% Tween 20 before incubation for 20 min at room temperature with horseradish peroxidase–conjugated goat antibodies to rabbit immunoglobulin G (1:2000 dilution) (Cell Signaling Technology). The membrane was again washed three times (5 min each time) with the same wash solution, after which immune complexes were visualized with the use of ECL reagents (GE Health Care, Chicago, IL). The gel was scanned with a VersaDoc Model 4000 imaging system (Bio-Rad) for measurement of band intensity.

### Statistical analysis

Data are presented as means ± SEM and were analyzed with Dunnett’s test or Tukey’s multiple comparison test. A *P* value of <0.05 was considered statistically significant.

## Results

### Effects of ω-3 LCPUFAs and lutein on CNV

**Fig 2:**
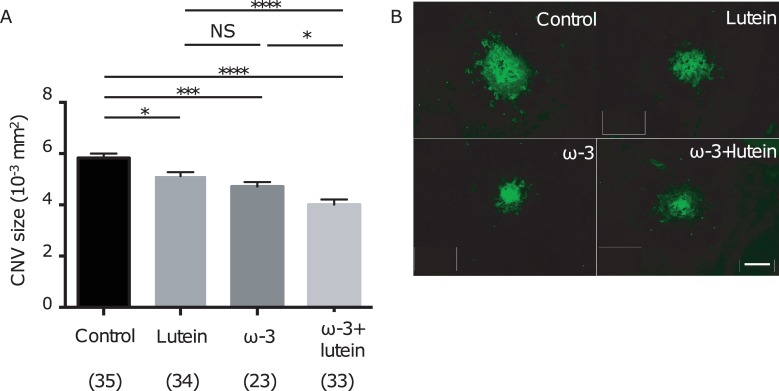
Effects of ω-3 LCPUFAs and lutein on the development of CNV. (A) The area of CNV at 7 days after laser photocoagulation was determined by staining of choroidal flat-mount preparations with Alexa Fluor 488–conjugated *G*. *simplicifolia* isolectin B4 for mice in the control group (*n* = 35 lesions), the ω-3 group (*n* = 23 lesions), the lutein group (*n* = 34 lesions), and the ω-3 + lutein group (*n* = 33 lesions). Data are means ± SEM. *P < 0.05, ***P < 0.001, ****P < 0.0001 (Tukey’s multiple comparison test). NS, not significant. The data shown in Fig 2A are from one of the three experiments performed. (**B**) Representative staining of CNV lesions for mice in the four groups. Scale bar, 100 μm.

To evaluate the effects of ω-3 LCPUFAs, lutein, and the combination of the two on the development of CNV, we fed mice with corresponding diets and administered lutein beginning 2 weeks and 1 week, respectively, before CNV induction by laser photocoagulation. Staining of choroidal flat-mount preparations with *G*. *simplicifolia* isolectin B4 revealed that the area of CNV at 7 days after laser photocoagulation was significantly smaller in mice receiving ω-3 LCPUFAs [(4.7192 ± 0.171) × 10^−3^ mm^2^] or lutein [(5.122 ± 0.172) × 10^−3^ mm^2^] compared with those not receiving either supplement [(5.828 ± 0.152) × 10^−3^ mm^2^] (Fig 2). Furthermore, these effects of ω-3 LCPUFAs and lutein on CNV size were additive [lesion area of (4.012 ± 0.189) × 10^−3^ mm^2^] (Fig 2). The general condition of mice including body size, coat condition, and appetite did not appear to differ among the four groups during the study period.

### Serum lipid and lutein profiles

**Fig 3:**
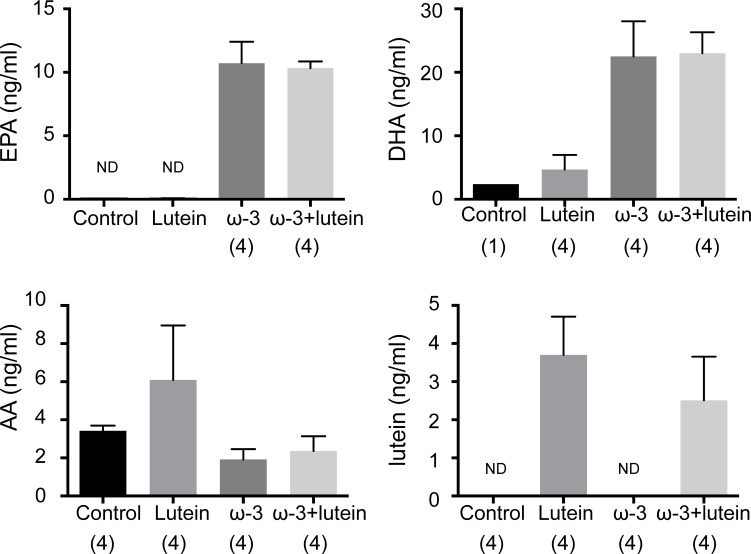
Serum concentrations of fatty acids and lutein. The serum levels of the ω-3 LCPUFAs EPA and DHA as well as those of AA and lutein were measured in mice of the four experimental groups at 7 days after laser photocoagulation. Data are means ± SEM (*n* = 4 mice per group). ND, not detected.

We analyzed lipid and lutein concentrations in serum collected from mice at 7 days after CNV induction (Fig 3). The serum levels of the ω-3 LCPUFAs eicosapentaenoic acid (EPA) and docosahexaenoic acid (DHA) were markedly increased in mice fed the ω-3 LCPUFA diet with or without lutein administration. Similarly, the serum concentration of lutein was greatly increased in mice receiving lutein. The serum level of arachidonic acid (AA), which replaced ω-3 LCPUFAs in the ω-3 LCPUFA–free diet, tended to be higher in mice fed the ω-3 LCPUFA–free diet than in those fed the ω-3 LCPUFA–containing diet, although these differences were not statistically significant (Dunnett’s test).

### Effects of ω-3 LCPUFAs and lutein on cytokine and chemokine concentrations in the retina and choroid

**Fig 4:**
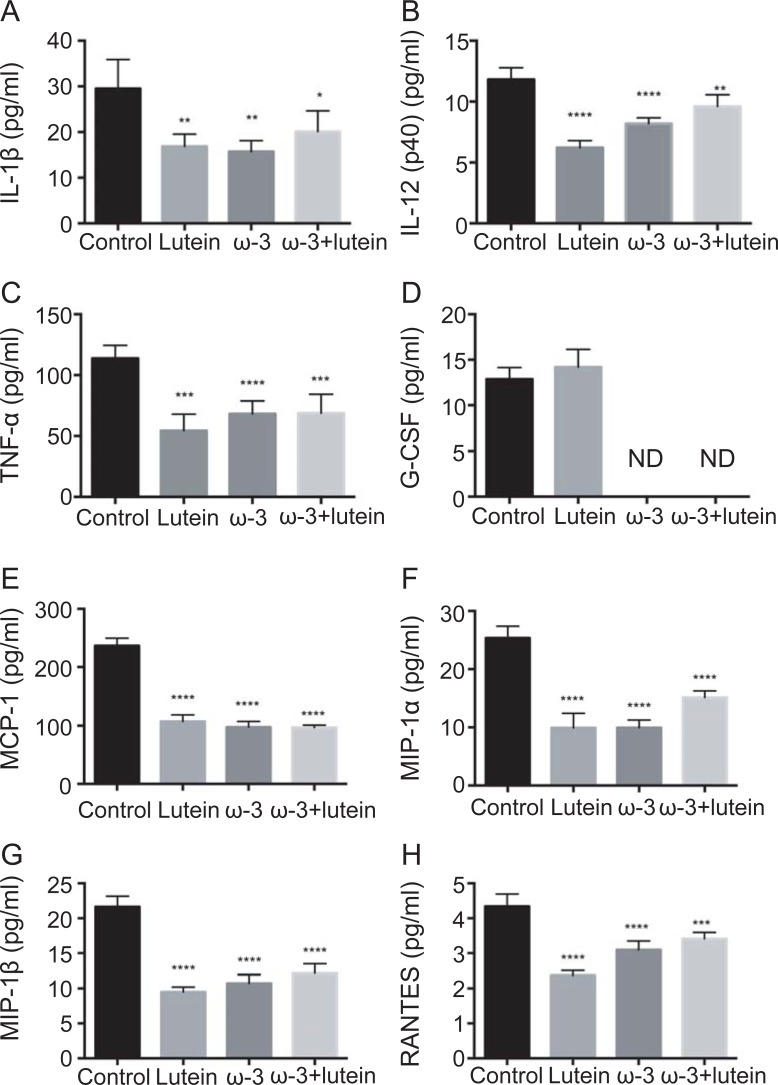
Effects of ω-3 LCPUFAs and lutein on the concentrations of representative proinflammatory cytokines and chemokines in the choroid. The concentrations of IL-1β (**A**), IL-12 (p40) (**B**), TNF-α (**C**), G-CSF (**D**), MCP-1 (**E**), MIP-1α (**F**), MIP-1β (**G**), and RANTES (**H**) in the choroid of mice in the four experimental groups were determined with a multiplex assay at 7 days after CNV induction. Data are means ± SEM of triplicate determinations for a pooled sample and are the same as those shown in S2 Fig. *P < 0.05, **P < 0.01, ***P < 0.001, ****P < 0.0001 versus the control group (Dunnett’s test). ND, not detected.

To investigate the mechanism by which the combination of ω-3 LCPUFAs and lutein attenuates CNV development, we measured the concentrations of inflammatory mediators in the retina and choroid at 7 days after laser photocoagulation with the use of a multiplex assay system (S1 and S2 Figs). The concentrations of the proinflammatory cytokines interleukin (IL)–1β, IL-12 (p40), and tumor necrosis factor–α (TNF-α) as well as those of the chemokines MCP-1 (monocyte chemoattractant protein–1), macrophage inflammatory protein (MIP)–1α, MIP-1β, and RANTES (regulated on activation, normal T expressed and secreted) in the choroid were significantly reduced in the lutein group, the ω-3 group, and the ω-3 + lutein group compared with the control group, whereas that of granulocyte colony-stimulating factor (G-CSF) was reduced to undetectable levels in mice fed the ω-3 LCPUFA diet (Fig 4). However, the concentration of none of these factors was reduced to a significantly greater extent in the ω-3 + lutein group than in the ω-3 or lutein groups.

### Effects of ω-3 LCPUFAs and lutein on oxidative stress in the retina

**Fig 5:**
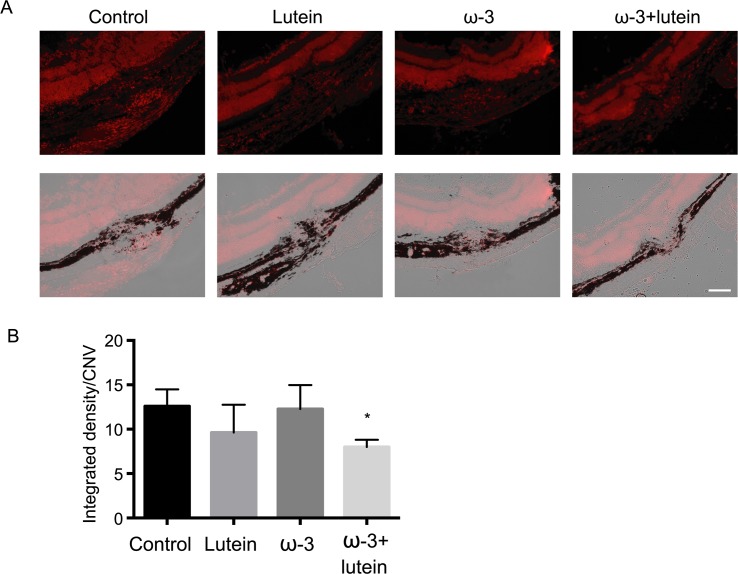
Effects of ω-3 LCPUFAs and lutein on ROS levels in RPE-choroid sections. (**A**) RPE-choroid sections prepared from mice in the four experimental groups at 5 days after laser photocoagulation were stained with DHE for detection of ROS. In lower panels, phase image was taken of corresponding DHE-stained images in upper panel, respectively. Scale bar, 100 μm. (**B**) Integrated density for DHE staining in the CNV area was measured with Image J software. Data are means ± SEM for four determinations of leasion of each experimental group. *P < 0.05 versus the control group (Dunnett’s test).

To investigate the effects of ω-3 LCPUFAs and lutein on oxidative stress induced by laser photocoagulation, we examined ROS levels in RPE-choroid sections by staining with DHE. DHE specifically reacts with superoxide and is thereby converted to the fluorescent compound ethidium. DHE staining intensity in retinal neural cells tended to be reduced in the ω-3 group and the lutein group compared with the control group, and these effects of ω-3 LCPUFAs and lutein were synergistic (Fig 5). The integrated density per CNV area was thus significantly smaller in the ω-3 + lutein group than in the control group (7.999 ± 0.402 versus 12.595 ± 0.945, with the values for the lutein and ω-3 groups being 9.631 ± 1.567 and 12.268 ± 1.354, respectively).

**Fig 6:**
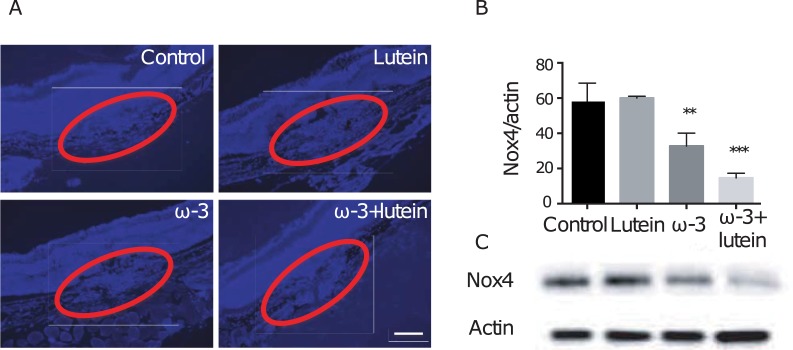
Effects of ω-3 LCPUFAs and lutein on Nox4 expression in the choroid-retina. (**A**) Representative images of Nox4 immunofluorescence (blue) in RPE-choroid sections prepared from mice of the four experimental groups at 7 days after laser photocoagulation. Red ovals enclose the lesion area. Scale bar, 100 μm. (**B**) The amount of Nox4 in the retina isolated from mice of the four experimental groups at 7 days after laser photocoagulation was determined by densitometric scanning of immunoblots. Data were normalized by the abundance of β-actin and are means ± SEM of triplicate determinations for a pooled sample. **P < 0.01, ***P < 0.001 versus control group. (**C**) Representative immunoblot of Nox4 and β-actin.

We also examined the expression of the ROS-generating enzyme Nox4 in the choroid and retina. Immunohistofluorescence staining revealed that the level of Nox4 immunoreactivity in the lesion area was reduced in mice of the ω-3, lutein, and ω-3 + lutein groups (Fig 6A). Immunoblot analysis of the retina revealed that the abundance of Nox4 was significantly reduced in the ω-3 group (32.8 ± 4.2), but not in the lutein group (60.0 ± 0.6), compared with the control group (58.1 ± 6.0) (Fig 6B and 6C). Furthermore, the amount of Nox4 was reduced synergistically in mice receiving both ω-3 LCPUFAs and lutein (14.7 ± 1.5).

## Discussion

We have here shown that dietary intake of ω-3 LCPUFAs and lutein in mice subjected to laser photocoagulation attenuated the development of CNV in an additive manner, and that this effect was accompanied by suppression of Nox4 expression and ROS production in the retina and choroid. The combination of ω-3 LCPUFAs and lutein thus likely relieves the oxidative stress that is thought to contribute to the abnormal vascular growth in this laser-induced AMD model. Our results suggest that further studies of dietary supplementation with ω-3 LCPUFAs and lutein are warranted in both patients with AMD and healthy individuals at risk for the development of this condition.

Dietary enrichment with ω-3 LCPUFAs such as DHA and EPA present in fish oil and certain plant and nut oils [2, 20, 21] has been shown to protect against inflammation [22, 23], pathological angiogenesis [24], and tumorigenesis [25–31]. We have previously shown that leukocyte recruitment to and adhesion molecule expression in choroidal neovascular lesions were down-regulated in mice fed ω-3 LCPUFAs, and that 17,18-epoxyeicosatetraenoic acid (EEQ) and 19,20-epoxydocosapentaenoic acid (EDP), the major metabolites of ω-3 LCPUFAs generated by cytochrome P450, were key mediators of disease resolution [18]. We have now shown that dietary ω-3 LCPUFAs reduced the level of oxidative stress in the retina of AMD model mice likely through down-regulation of Nox4 expression. Lutein is the major dietary xanthophyll in humans and an abundant carotenoid in nature [32, 33]. Both carotenoids and fatty acids are components of the cell membrane, with interactions between these molecules having been shown to modulate various cellular and physiological processes [34]. We found that the antioxidative effect of dietary ω-3 LCPUFAs in mice subjected to laser photocoagulation was enhanced by intragastric oral administration of lutein in a synergistic manner.

Various factors such as sunlight exposure, diet, smoking, and vitamin D levels [35–37] have well-documented effects on oxidative stress, are proinflammatory, and promote the progression of early AMD drusen to CNV or geographic atrophy [38]. ω-3 LCPUFAs have also previously been found to suppress oxidative stress. DHA was thus shown to induce nuclear translocation of the oxidative stress sensor NFE2L2 and thereby to trigger an antioxidative response in RPE cells [39] and cancer cells [40]. We have now shown that ω-3 LCPUFAs act synergistically with lutein to reduce oxidative stress in a mouse model of CNV, although the mechanism responsible for this synergism remains unknown. The bioavailability of carotenoids depends on their bioaccessibility, which is in turn influenced by factors such as the incorporation of additional lipids during or after their processing, structural barriers including food matrix characteristics, and dietary fiber content [41, 42]. For instance, the addition of extra oil is known to improve the bioavailability of carotenes, which is dependent on their distribution and solubilization in micelles (dissolution) rather than on ester hydrolysis [43]. In the present study, we used marigold-derived esterified lutein and administered it with oral gavage needle. Administration of lutein with or without ω-3 LCPUFAs increased the serum lutein concentration in mice to similar extents, indicating that lutein is absorbed into the circulation and is then able to act synergistically with ω-3 LCPUFAs to reduce oxidative stress in chorioretinal lesions of CNV.

With regard to the possible role of cytokines or chemokines in the additive or synergistic effects of ω-3 LCPUFAs and lutein on chorioretinal lesions, the retinal or choroidal concentrations of none of the 23 such mediators examined were reduced to a significantly greater extent in response to the combination of ω-3 LCPUFAs and lutein than in response to either ω-3 LCPUFAs or lutein alone. Mice fed ω-3 LCPUFAs, lutein, or both ω-3 LCPUFAs and lutein showed significantly reduced concentrations of IL-1β, IL-12 (p40), TNF-α, MCP-1, MIP-1α, MIP-1β, and RANTES in the choroid. These results thus suggest that dietary intake of ω-3 LCPUFAs plus lutein indeed suppresses the inflammatory response in the choroid of mice with laser-induced CNV, but that this action does not contribute to the synergistic or additive effects of these dietary agents on oxidative stress and CNV development.

## Supporting information

S1 Fig Concentrations of proinflammatory cytokines and chemokines in the retina. The concentrations of 23 cytokines and chemokines in the retina of mice in the four experimental groups at 7 days after laser photocoagulation were determined with a multiplex assay. Data are means ± SEM of triplicate determinations for a pooled sample. IL-3, IL-4, IL-5, IL-6, IL-9, IL-10, IL-12 (p70), IL-17A, eotaxin, granulocyte-macrophage (GM)–CSF, interferon-γ (IFN-γ), and keratinocyte chemoattractant (KC) were not detected.
(EPS)

S2 Fig Concentrations of proinflammatory cytokines and chemokines in the choroid. The concentrations of 23 cytokines and chemokines in the choroid of mice in the four experimental groups at 7 days after laser photocoagulation were determined with a multiplex assay. Data are means ± SEM of triplicate determinations for a pooled sample. IL-3, IL-4, IL-5, IL-10, IL-12 (p70), IL-13, IL-17A, eotaxin, GM-CSF, and KC were not detected.
(EPS)
